# Staining Invariant Features for Improving Generalization of Deep Convolutional Neural Networks in Computational Pathology

**DOI:** 10.3389/fbioe.2019.00198

**Published:** 2019-08-23

**Authors:** Sebastian Otálora, Manfredo Atzori, Vincent Andrearczyk, Amjad Khan, Henning Müller

**Affiliations:** ^1^Institute of Information Systems, HES-SO University of Applied Sciences and Arts Western Switzerland, Sierre, Switzerland; ^2^Computer Science Centre (CUI), University of Geneva, Geneva, Switzerland; ^3^Institute of Pathology, University of Bern, Bern, Switzerland; ^4^Medical Faculty, University of Geneva, Geneva, Switzerland

**Keywords:** staining normalization, adversarial neural networks, digital pathology, color augmentation, color normalization, domain shift

## Abstract

One of the main obstacles for the implementation of deep convolutional neural networks (DCNNs) in the clinical pathology workflow is their low capability to overcome variability in slide preparation and scanner configuration, that leads to changes in tissue appearance. Some of these variations may not be not included in the training data, which means that the models have a risk to not generalize well. Addressing such variations and evaluating them in reproducible scenarios allows understanding of when the models generalize better, which is crucial for performance improvements and better DCNN models. Staining normalization techniques (often based on color deconvolution and deep learning) and color augmentation approaches have shown improvements in the generalization of the classification tasks for several tissue types. Domain-invariant training of DCNN's is also a promising technique to address the problem of training a single model for different domains, since it includes the source domain information to guide the training toward domain-invariant features, achieving state-of-the-art results in classification tasks. In this article, deep domain adaptation in convolutional networks (DANN) is applied to computational pathology and compared with widely used staining normalization and color augmentation methods in two challenging classification tasks. The classification tasks rely on two openly accessible datasets, targeting Gleason grading in prostate cancer, and mitosis classification in breast tissue. The benchmark of the different techniques and their combination in two DCNN architectures allows us to assess the generalization abilities and advantages of each method in the considered classification tasks. The code for reproducing our experiments and preprocessing the data is publicly available^1^. Quantitative and qualitative results show that the use of DANN helps model generalization to external datasets. The combination of several techniques to manage color heterogeneity suggests that several methods together, such as color augmentation methods with DANN training, can generalize even further. The results do not show a single best technique among the considered methods, even when combining them. However, color augmentation and DANN training obtain most often the best results (alone or combined with color normalization and color augmentation). The statistical significance of the results and the embeddings visualizations provide useful insights to design DCNN that generalizes to unseen staining appearances. Furthermore, in this work, we release for the first time code for DANN evaluation in open access datasets for computational pathology. This work opens the possibility for further research on using DANN models together with techniques that can overcome the tissue preparation differences across datasets to tackle limited generalization.

## 1. Introduction

Since its start, one of the main goals of computational pathology (CP) is to find precise and reproducible methods to quantify the content of tissue slides and the relationships of this with the disease stage and patient outcome (Madabhushi, 2009; Madabhushi et al., 2011; Al-Janabi et al., 2012; Kothari et al., 2013). During the last decade the methods for analyzing images in digital pathology have become more precise and have greatly benefited the CP community, also thanks to the steady development of deep learning algorithms and particularly thanks to deep convolutional neural networks (DCNN), Shifting from handcrafted features toward end–to–end architectures [to detect cancer in histopathology images at the image patch level (Veta et al., 2015; Janowczyk and Madabhushi, 2016; Ciompi et al., 2017) and at the whole-slide-image level (Litjens et al., 2016; Cruz-Roa et al., 2018)], methods have become more precise, achieving is some cases for specific tasks classification performance comparable to pathologists.

Despite the performance improvements of the methods, there are still technical barriers that prevent the translation of these advances into better clinical applications. Two of the most typical chemicals used in pathology to stain tissue slides are Hematoxylin and Eosin. These chemicals highlight the nuclei with a dark purple color (Hematoxylin) and the cytoplasm with a light pink one (Eosin). One of the most important factors preventing the application of machine learning methods to clinical practice is related to the heterogeneity of Hematoxylin and Eosin (H&E) images due to tissue preparation and several parameters involved in the tissue preparation and digital scanning process (temperature of the tissue, thickness of the cuts, image sensor of the digital camera, etc.). Several image processing and machine learning techniques reported in the literature deal with color heterogeneity improving classification and segmentation performance for various tissue types (Van Eycke et al., 2017; Roy et al., 2018; Tellez et al., 2019). However, this challenging problem is far from being solved.

**Figure 1:**
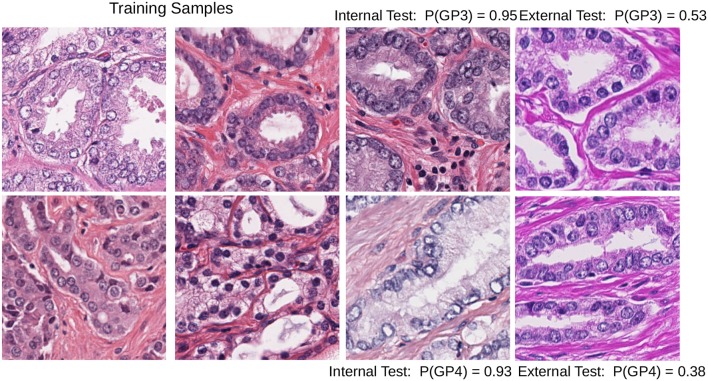
Test images with different staining conditions can affect the performance of a DCNN model trained with images with a limited set of similar staining and preparation methods: Gleason pattern 3 (top row) and pattern 4 (bottom row) patches; the internal test set probability (third column) can lead to biased estimates of the performance of the model. The last column shows how probability drops in the baseline DCNN when predicting the class in patches with different staining.

Color heterogeneity can affect the performance of the machine learning algorithms that easily overfit when trained with data from one center or scanner and fail to generalize to images from other centers (Kothari et al., 2014; Ciompi et al., 2017). One example of this problem is shown in Figure 1.

The heterogeneity of colors in H&E images is due to tissue preparation and is related to the complex set of preparation phases related to staining procedures, section thickness, and scanner differences (Leo et al., 2016).

One of the more recurrent problems in the preparation of the specimen is over- or under-staining. The time and amount of dye applied to the tissue on the glass make the process prone to perceptual differences in color and intensity of the tissue in the digitized slide (Bejnordi et al., 2016; Van Eycke et al., 2017).

The changes in color intensity due to different slice thicknesses are acknowledged as a source of variation. Few articles have explored how to overcome this aspect using automatic tools, presumably because of the complexity of the experimental setups. Notably, the work of Bug et al. (2017) provides a staining normalization method based on an end-to-end deep learning (DL) architecture that takes into account the context of the tissue to normalize the extracted features. The authors evaluated their method on lung cancer tissue images varying not only in terms of H&E concentrations but also in section thickness. They showed that their method gives a consistent normalization through the studied protocols and less variance in the output with respect to the classic methods of Macenko et al. (2009) and Bejnordi et al. (2016).

Although standardization procedures are often applied in laboratories and clinical practice, perfect color calibration among samples is hard to achieve. The scanning parameters that are hardcoded in the whole slide scanner hardware (that change according to the vendors, such as Aperio, Philips, Ventana) result in specific color characteristics in the resulting digital image, for example, varying in the image sensor and also the stitching techniques. These parameters are also a part of the digital pathology pipeline on which pathologists have limited control. In the work of Leo et al. (2016) the authors evaluate the stability of features in prostate cancer classification across several scanner producers. Their results show that only a portion of the commonly used features in digital pathology are robust to such scanner differences. Interestingly, the authors show that color normalization alone cannot solve the problem of inter-scanner feature instability. Therefore, it is crucial to develop algorithms that generalize well on heterogeneous datasets for a more robust deployment of computer-aided diagnostic systems (Kothari et al., 2013; Leo et al., 2016).

Image processing and machine learning approaches that deal with color heterogeneity show generalization improvements in classification and segmentation performance (McCann et al., 2014; Vahadane et al., 2016; Bentaieb and Hamarneh, 2018; Ren et al., 2018; Roy et al., 2018; Tellez et al., 2018a,b, 2019). Such techniques can generally be grouped into three different types. The first approach (which is probably the most frequently studied) is staining normalization, meaning that the color concentration of all the images in the database is mapped to match the staining appearance of a target image. This problem has been thoroughly studied in literature (Macenko et al., 2009; Li et al., 2015; Ciompi et al., 2017; Van Eycke et al., 2017). Staining normalization applied with DCNN allowed to improve classification accuracy by over 20% (Ciompi et al., 2017) in colorectal cancer tissue classification. In the work of Van Eycke et al. (2017) a series of steps for color vector extraction is described. Two main challenges often arise when using staining normalization techniques. First, as the source images are color-transformed into the target image color space, the algorithms using the normalized images are sensitive to the selection of the image taken as reference (Bentaieb and Hamarneh, 2018). Second, the time of staining normalization algorithms for standardizing a batch of images can easily take up to 30 minutes as noted by Shaban et al. (2018).

The second approach is color augmentation, where many variations of the original image are created for training by varying intensity, brightness, contrast, RGB channel values and also altering them in other color spaces such as the Hue Saturation Value (HSV) space, or deconvolving the Hematoxylin-Eosin-DAB (HED) channels and slightly modifying them (Van Eycke et al., 2018; Bandi et al., 2019; Tellez et al., 2019). The color augmented images are usually generated at training time and given as input to the algorithm to cope with possible variations in the test set. Data augmentation is usually built into the pipeline of training deep learning models, since such models usually learn color invariances by processing a large amount of annotated samples. Color augmentation can lead to excellent results, such as the ones obtained by the leading teams of the Camelyon17 challenge (Bandi et al., 2019). These approaches use deep learning architectures in conjunction with extensive color augmentation to force their networks to be robust to color variation.

The third and most recent family of techniques is inherently related to deep learning architectures. Deep learning models account for the complexity of learning the staining transformation in the test set by means of learning a cascade of non-linear transformations of the training input images. This learnt information is used to either normalize the image or to explicitly design a deep learning model architecture that penalizes or does not take into account the staining information (Bug et al., 2017; Janowczyk et al., 2017; Ren et al., 2018). This deep learning-based family of techniques is further discussed in section 2.4.

As discussed, staining variability and color augmentation can dramatically affect automatic image analysis algorithms and several techniques have been proposed to tackle the problem depending on the image analysis task. Perfect color calibration among samples is hard to achieve, despite standardization procedures being applied in clinical practice. In this paper, we contribute to tackle this challenge by comparing the three mentioned approaches in intra and inter–center classification tasks (targeting the classification of mitotic cells in breast cancer and Gleason pattern classification in prostate cancer). We thoroughly evaluate the adversarial neural network training approach first proposed by Lafarge et al. (2017) to learn domain invariant features, showing that the use of DANNs can help generalization to external datasets. The combination of techniques also suggests that by using basic color augmentations in addition to techniques such as DANN training and staining normalization, the models can generalize even further.

## 2. Materials and Methods

### 2.1. Color-Heterogeneous Datasets

For comparing the generalization capabilities of the approaches, we used two highly heterogeneous data sets that target classification tasks. The data sets account for the staining variability by including images with differing preparation parameters from several centers. Such parameters result in strong differences in the visual characteristics of the images.

Most frequently, for the evaluation of algorithms, subsets of images from the same center are used as a training set and then validation and testing is done with images of different patients but with similar image preparation characteristics from the same center.

In our evaluation, we extend the classical evaluation by separating internal test data: different patients with similar preparation parameters, from external test data: different patients and preparation characteristics. Such an evaluation provide us with a better approximation of the performance of the algorithms on images acquired from different centers, i.e., the generalizability of the networks to such changes.

**Figure 2:**
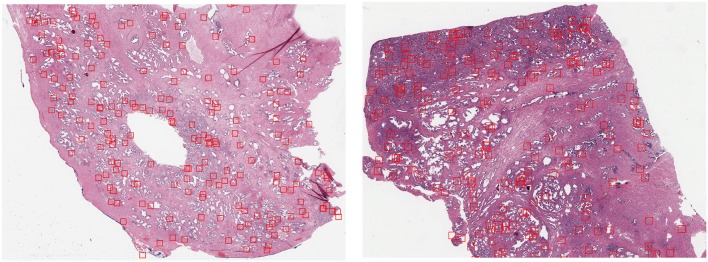
GP3 patch locations extracted (red bounding boxes) from slide TCGA-2A-A8VL **(Left)** belonging to the training set and the slide TCGA-EJ-7321 **(Right)** from the internal test set using the heatmap resulting from Equation (2).

**Figure 3:**
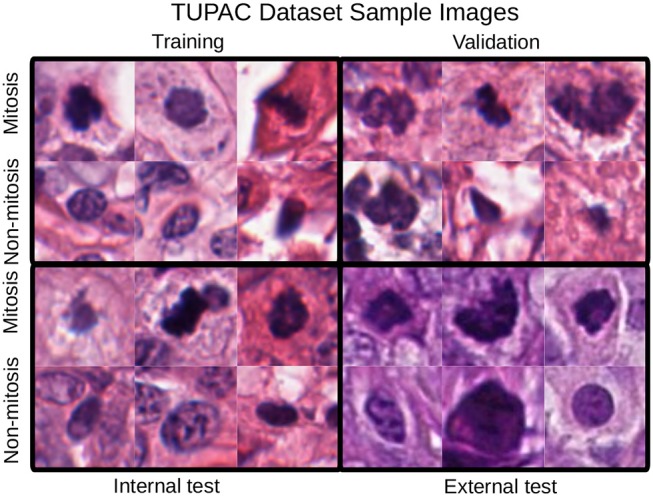
Mitotic figure examples with an original patch size of 96 × 96 pixels. Staining differences between the internal and the test sets are evident. These changes are also noticeable in the quantitative results of section 4.

The first dataset is the Tumor Proliferation Assessment Challenge (TUPAC), which is built to evaluate algorithmic performance for mitotic figure detection in breast cancer tissue (Veta et al., 2019). We refer to this dataset as TUPAC. The dataset contains 1,522 mitotic figures extracted from high power fields of 73 breast cancer cases from three pathology labs. This suits our purpose of evaluating the generalization abilities of algorithms across inter-center variability. The dataset partition is the same as in Lafarge et al. (2017). All our DCNN models in this dataset are trained and validated with eight (458 mitoses) and four cases (92 mitoses) from the first pathology lab. The remaining 12 cases (533 mitoses) from the first pathology lab are used as an internal test set. The 50 examples from the two other pathology labs (469 mitoses) are used as an external test set. To create a set of challenging negative samples, an initial CNN is trained with mitotic patches as positive patches and on random patch locations as negative ones that do not overlap with mitotic patches. Once the network is trained, only the false positives and negative patches with a high probability of being mitotic are taken as negative samples, this set of hard-negative mined samples are kept throughout all the experiments. Our hard-negative mining differs, and thus, the performance is not directly comparable with those reported by Lafarge et al. (2017). The cardinality of each partition is in Table 1, and a few example image locations are shown in Figure 2 for TCGA-PRAD and example image patches in Figure 3 for the TUPAC dataset, respectively.

**Table 1:** Number of original patches for the TUPAC dataset, due to the high class imbalance, we use data augmentation only in the mitotic class by creating rotated and flipped patches.

**Partition**	**Mitosis**	**Non-mitosis**	**Total**	**# of cases**	**Center**
Train	458	3,842	4,300	8	1
Validation	92	1,196	1,288	4	1
Internal test	533	12,317	12,850	12	1
External test	469	505	974	50	2,3
Total	1,552	17,860	19,412	74	2,3

**Figure 4:**
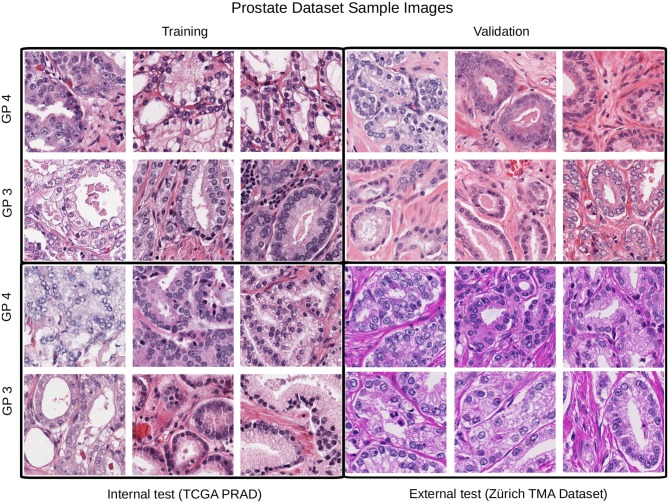
Example patches from each partition for the Gleason pattern classification task. In this case the external test set differs considerably from the training, validation, and test partitions.

The second dataset is comprised of images from prostate cancer tissue with Gleason patterns 3 and 4 (GP3, GP4). It contains image patches from diagnostic slides of the cancer genome atlas prostate adenocarcinoma dataset (TCGA-PRAD) and also from the manually annotated prostate tissue microarray images used in the study of Arvaniti et al. (2018), we refer to this dataset as TCGA-TMAZ. The TCGA-PRAD images were recorded in 16 tissue source centers^2^ that were used for training, validation and internal test sets. Whereas, the TMA-Zürich where used as an external test set since it contains significant staining variability as shown in Figure 4.

Since in the TCGA-PRAD dataset there are no Gleason pattern annotations but only the global labels of the two most prominent Gleason patterns, we selected only those diagnostic images with the same primary and secondary Gleason patterns, i.e., GS = 3 + 3 and GS = 4 + 4. With this consideration, we are more likely to extract patches from relevant regions. Furthermore, we employ the heuristic first presented by Rousson et al. (2018), to build a heatmap that guides patch extraction from non-annotated Whole Slide Images (WSI) using the following transformation of the original RGB WSI at the 10X magnification level:

$$\begin{array}{rcl} h_{1} & = & \tanh\left(\frac{2\left(B-R\right)}{G+1}+0.5\right)+0.5; \end{array}$$

(1) $$\begin{array}{rcl} h_{2} & = & \tanh\left(\frac{640-R-G-B}{300}+0.5\right)+0.5 \end{array}$$

(2) $$\begin{array}{r} h=0.5\tanh\left(h_{1}h_{2}-1.75\right)+0.5 \end{array}$$

By fixing a threshold (0.65) on the resulting heatmap *h* we selected a fixed amount of random patches in the WSI from positive locations in the thresholded image. Example locations of extracted patches from two WSIs of the train and internal test sets are illustrated in Figure 2. While there are a minority of patch locations outside relevant regions, this unsupervised approach to locate regions of interest allows us to have the right amount of relevant regions that contains GP3 and GP4 patches.

In contrast to the TUPAC dataset, in this case, we have more than 3 centers, the total number of source centers for the TCGA-TMAZ dataset is 34, which makes it challenging to train the algorithms with a limited amount of images for each center. The total number of images per partition is reported in Table 2.

**Table 2:** Number of original patches for the TCGA-TMA dataset.

**Partition**	**GP3**	**GP4**	**Total**	**#centers**
Train	1,184	1,219	2,403	6
Validation	479	658	1,137	4
Internal test	811	510	1,321	6
External test	1,602	2,359	3,961	1
Total	4,076	4,746	8,822	17

We do not have a larger public data set with information on the different centers, which is a current challenge for evaluation of the generalization of CP algorithms. In the literature there are interesting approaches to solve CP classification tasks that use more than 100 million annotated patches as in Nagpal et al. (2018). However, it is noteworthy that in this work we did not use any annotations of regions or private data source. Only the public annotations of the TUPAC mitotic regions and an open access dataset with limited annotations are used. The scripts and filename lists with (x,y) locations of the patches are provided in order to allow reproducing the same image patches used in our experiments.

### 2.2. Staining Normalization

Since all the pixels of the digital H&E images are represented in RGB space, ideally each pixel should contain a composition of the color representation of Hematoxylin, Eosin, and background. Images acquired from the same center and using the same preparation parameters should share the stain absorbance coefficients, which can be written as the linear transformation (omitting background that should be close to 255 for the three channels):

$$S=\left(\begin{array}{ccc} H_{R} & H_{G} & H_{B}\\ E_{R} & E_{G} & E_{B} \end{array}\right)$$

Where the first-row vector corresponds to the RGB components of hematoxylin and the second one to the components of Eosin. In staining normalization methods, the aim is to estimate the individual staining absorbance coefficients of the images *S* and quantify the absorbed light **C** by the tissue when it was scanned, which is the value in the H&E space of each pixel. The Beer-Lambert law provides a way to estimate them in the optical density space, given the original pixel content for the *c*-channel *I*_*c*_:

$$I_{c}=I_{0}\exp\left(-S_{c}\cdot \text{C}\right)$$

**Figure 5:**
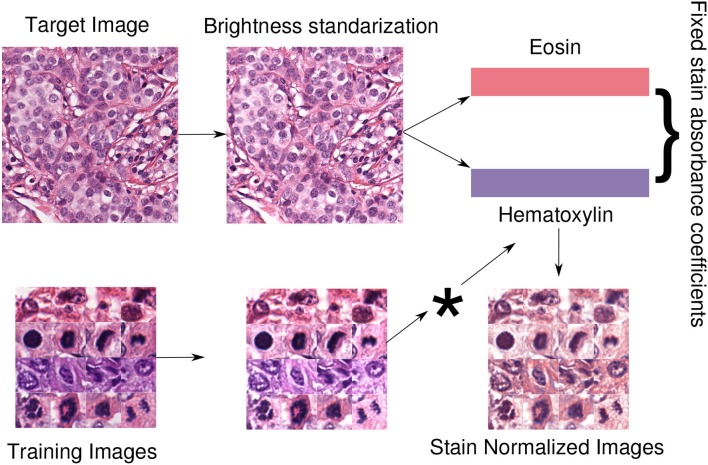
Staining normalization scheme. First, a target or template image is selected to extract its staining concentrations. With brightness normalization, the images are less dependent regarding brightness. Therefore, brightness standardization is done by modifying the luminosity channel in the LAB color space such that at least 5% of the pixels are white. Then, the staining concentration matrix from the brightness-corrected template image is extracted using the Macenko method (Macenko et al., 2009). Finally, all the images in the dataset are normalized using the fixed template staining (*indicates pixelwise multiplication with the template).

Where *c* ranges in the RGB channels, *S* ∈ [0, +∞]^3×2^ is the matrix of absorbance coefficients, **C** ∈ [0, +∞]^2^ is the vector of the two staining concentration coefficients and *I*_0_ is the background value. The widely used method of Macenko et al. (2009) provides an estimation of *S* by computing a plane using the two largest singular value decomposition vectors of the image and then projecting the data into this plane and clipping extreme values. Using this estimation, the matrix *S* is fixed to normalize all the images in the database by multiplying each value of the concentration of the source images pixelwise. Then, the image is in the H&E space of the target (template) image. An optional step is to preprocess the image with brightness standardization making the obtained coefficients less dependent on the brightness values. A schematic view of the normalization approach, used in our experiments, is displayed in Figure 5.

### 2.3. Color Augmentation

One common strategy used to create new images with color variations consists of multiplying each of the color channels *I*_*c*_ (or estimated staining concentrations in the H&E space) of the original image by a random small constant *a*_*c*_ that will scale the original intensity value and then add a second constant *b*_*c*_ that shifts the color toward higher/lower intensity values:

(3) $$\begin{array}{r} I_{c}^{\prime }\leftarrow a_{c}I_{c}+b_{c} \end{array}$$

**Figure 6:**
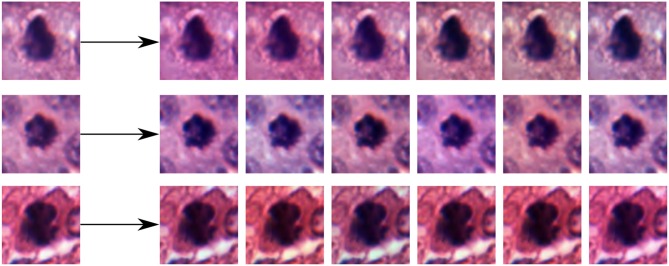
Examples of random color augmentations for training patches induced by Equation (3).

The range from where the random constants *a*_*c*_ and *b*_*c*_ are drawn determines how much variation is allowed in the generation of the new images. Examples of color augmented patches from the TUPAC dataset using this strategy in the RGB space are shown in Figure 6.

While staining normalization methods aim at homogenizing the appearance of the images, they might fall short at inter-center generalization because of a non-optimal normalization or overfitting in the training set due to the small amount of variation between the samples. Recent studies have shown how by creating images for training with an expansion of the training set with duplicate images from a broader color range have a significant impact at generalization in the inter-center evaluation of machine learning methods. Particularly with deep learning architectures, studies have shown how color augmentation techniques usually outperform staining normalization methods (Bejnordi et al., 2017; Tellez et al., 2019). This might be intuitive in the context of training deep learning models, where the larger the amount of data the model is fed with, the more variations the model is exposed to. Therefore, it is more robust to changes in appearance in the test set.

### 2.4. Deep Learning Approaches for Staining Normalization

Recent approaches based on deep learning models such as staining normalization stacked autoencoders (Janowczyk et al., 2017) and U-net-based architectures (Tellez et al., 2019) allow to capture complex staining transformations and build normalizers and generators in an end-to-end manner. In the work of Tellez et al. (2019) the impact of color augmentation and staining normalization is assessed in three organs regarding cancer: prostate, breast, and colorectal. The authors propose a normalization method based on a U-Net architecture that mapped augmented versions of the image to a normalized one. This showed significantly better results than standard augmentation and normalization methods. In Bentaieb and Hamarneh (2018), the authors explored several ways of training a deep adversarial staining transfer model in colon, ovary and breast cancer datasets showing that a combination of penalty terms of consistency of the normalized images and a conditional term that adapts to the task leads to the best results in classification tasks. The models mentioned above require the training of an external deep learning model that normalizes the images. This is used subsequently for the DCNN model training of the task, thus not using the source domain information for guiding the training of staining invariant features.

### 2.5. Domain Adaptation and Adversarial Learning

From a data-driven perspective, the disparities between the digital histopathology image stainings caused by the changes in preparation methods and scanners make the distribution of the generated images differ. The training of machine learning models with only images created from a subset of preparation methods, scanners or centers creates vulnerable models that might fail at correctly classifying near out-of-distribution samples with staining changes. Discriminative learning methods for classification, such as neural networks or support vector machines, perform well when training and test data are drawn from the same distribution. Therefore, when a model is trained using a set from one distribution and then tested in another, its performance will be hindered by the dissimilarity between the training and test distributions. This is a well-known problem in machine learning called domain adaptation (DA). It is an active research area in machine learning where many novel frameworks have been proposed (Crammer et al., 2006; Ben-David et al., 2007; Tzeng et al., 2017).

In Ben-David et al. (2007), the authors provided a framework to analyze the contributions of domain adaptation for the generalization of models by learning features that account for the domain disparity between training and test set distributions. The theory of DA suggests that a good representation for cross-domain transfer is one in which the algorithm cannot learn to identify the domain of origin of the input observation. This led to the authors of Ganin et al. (2016) to propose a concrete implementation of this idea in the context of deep neural network models. The objective of domain adversarial neural network models (DANN), is to learn features that do not take into account the domain of the training samples. The domain adversarial features combine discriminativness and domain invariance into the same representation. To build such representations, two classifiers are involved: (i) the label classifier that predicts the main task classes (e.g., mitosis/non-mitosis) at training and testing time, and (ii) the domains classifier that discriminates between several domains. Both classifiers can be tied using the same set of features. Having two outputs with separate loss functions, one that measures the error at classifying the class of the sample correctly and the second one,measuring the error at classifying the origin of the sample (i.e., the domain).

The optimization objective of a DANN model is to find a saddle point solution of parameters for the task classifier ${\hat{\theta }}_{y}$, domain parameters ${\hat{\theta }}_{d}$ and domain-invariant features ${\hat{\theta }}_{f}$. The loss function for the *N* = *n* + *n*′ training samples is:

$$L=\frac{1}{n}\overset{n}{\underset{i=1}{\sum }}L_{y}^{i}\left(\theta_{f},\theta_{y}\right)-\lambda \left(\frac{1}{n}\overset{n}{\underset{i=1}{\sum }}L_{d}^{i}\left(\theta_{f},\theta_{d}\right)+\frac{1}{n^{\prime }}\overset{N}{\underset{i=n+1}{\sum }}L_{d}^{i}\left(\theta_{f},\theta_{d}\right)\right)$$

where $L_{y}$ and $L_{d}$ are the task and domain loss functions, respectively. Both *n* and *n*′ are drawn from the dataset with the difference that *n* are training samples for which the task label is available, where for the *n*′ samples only the domain is known. It is worth noting that *n*′ are the samples with different domains in the training but can include samples from the test set, from which only the domain label is needed to adapt the shared feature representation. In Ganin et al. (2016) the authors find that such saddle points can be found using the following iterative stochastic gradient updates:

(4) $$\begin{array}{r} \theta_{f}\leftarrow \theta_{f}-\mu \left(\frac{\partial L_{y}^{i}}{\partial \theta_{f}}-\lambda \frac{\partial L_{d}^{i}}{\partial \theta_{f}}\right), \end{array}$$

(5) $$\begin{array}{r} \theta_{y}\leftarrow \theta_{y}-\mu \frac{\partial L_{y}^{i}}{\partial \theta_{y}}, \end{array}$$

(6) $$\begin{array}{r} \theta_{d}\leftarrow \theta_{d}-\mu \lambda \frac{\partial L_{d}^{i}}{\partial \theta_{d}} \end{array}$$

Where μ is the base learning rate for the task classifier and λ is the domain learning rate multiplier that allow to learn the domain-dependant features at a different pace from the task-related ones. Equations (5) and (6) optimize the parameters for the task and domain loss functions, respectively, as in a classical multi-output DCNN scenario. The loss for the task classifier is minimized while the loss for the domain classifier is maximized. This is done in Equation (4) that is optimized in an adversarial manner by going in the opposite gradient direction that minimizes task loss and in the positive direction of the gradient for the domain features, maximizing the domain classifier loss.

**Figure 7:**
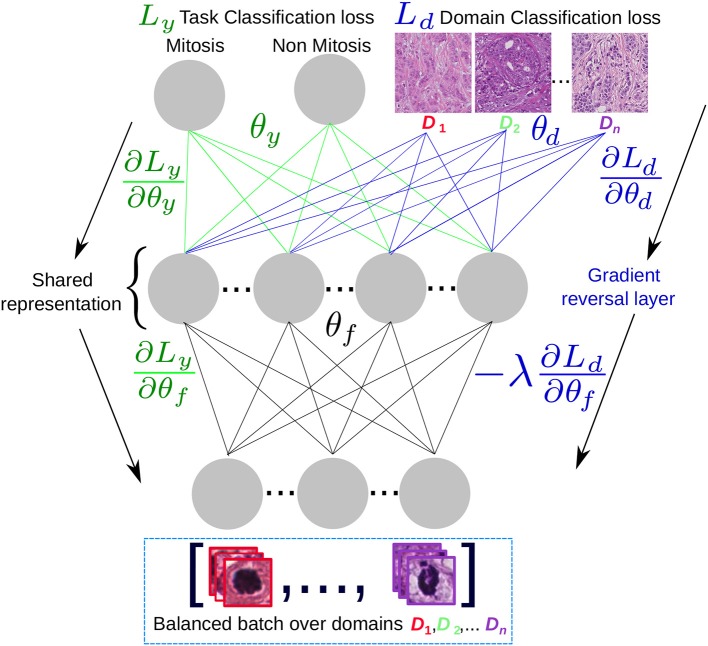
Domain adversarial scheme: A domain-balanced batch of images is passed as input to the network that has two types of outputs: the task classification output and the domain classification output. The shared representation θ_*f*_ is optimal for the task classification and unable to discriminate between the *n* domains.

A simplified architecture scheme is shown in Figure 7, where the task and the domain classifiers are at the top of a shared representation that maximizes the classification performance to separate between mitotic and non-mitotic images and at the same time minimizes the probability to recover in which center (domain) the image was generated. If the model converges, then for a test image the inferred representation avoids to include any center-specific information. The original center for the test image could be unknown, since this label is not needed and we are only interested in obtaining the mitosis/non-mitosis probability.

The main characteristic of DANN models is that they do not require external models to perform staining normalization or color augmentation but are the task labels and the indicators from the scanners or domains that are used to drive the training process and solve the task without depending explicitly on such models. This feature is also a potential drawback if one is interested in a staining quantification scenario, because DANN models do not output an explicit normalized image but the normalization is done in the feature space instead. This group samples from the same class regardless of the domain from where they were generated. Our experiments are inspired by the work of Lafarge et al. (2017) where the authors build a DCNN with a reverse gradient layer that aims to learn in an adversarial manner the mitotic probability of patches and their domain.

It was noted by Engilberge et al. (2017) that in the context of DCNN, the models are sensitive to color changes and also that in the more common architectures, the color-sensive units are located in the first layers of the network, which suggests that the stain-dependent information of the network should be removed from the first layers of the architectures, this lead us to place the reversal gradient information before the fully connected layers of the network as shown in the experimental setup section 3.

## 3. Experimental Setup

### 3.1. CNN Architectures

We designed a baseline DCNN model for both datasets. For the TUPAC dataset, we designed a relatively compact DCNN architecture due to the small input size of 96 × 96 × 3 patches. The architecture is composed of two convolutional layers followed by two blocks of convolution, batch normalization, and max-pooling. Then, a dropout layer is included to regularize the model followed by another block of convolution, batch normalization, and max-pooling. At the end of the network, two branches of dense layers are connected to the mitosis and domain class predictions. For the domain branch, the gradient is reversed in order to optimize (Equation 4).

For the TCGA-TMAZ dataset, we used as base model the MobilNet architecture used in the experiments by Arvaniti et al. (2018). The last layer was removed in order to add the two branches for the domain and Gleason pattern classification. The gradient reversal layer was added in the same way as in the TUPAC architecture, i.e., after the common convolutional filters and before the domain-specific dense features.

### 3.2. Implementation Details

We implemented the DCNN architectures and performed model training using the Keras deep learning framework with the Tensorflow backend. For the staining normalization method, we used the public implementation of the Macenko method from the StainTools library^3^. A crucial implementation detail is the need to generate domain-balanced batches for the adversarial update of DANN models; this is a dataset-specific part of the pipeline where the number of domains should be provided, since they are used as a one-hot encoding domain-label vector for each of the samples. The learning rates were explored in the base models and fixed to μ = 0.01 for the mitosis model and μ = 0.001 for the Gleason pattern classification model, according to the best results on the test set.

A warmup of 100 batch iterations for the task branch (λ = 0) in the DANN model was observed to lead to a more stable training as compared to starting with random weights for both branches. We provide the code of our experiments for further implementation details^4^.

Four DCNN models were trained for each experiment combination, to minimize performance variations in the test sets due to the random-weight initializations of the DCNN models. Average and standard deviation in performance is the final result reported. In total 56 DCNN models were trained.

## 4. Results

**Figure 8:**
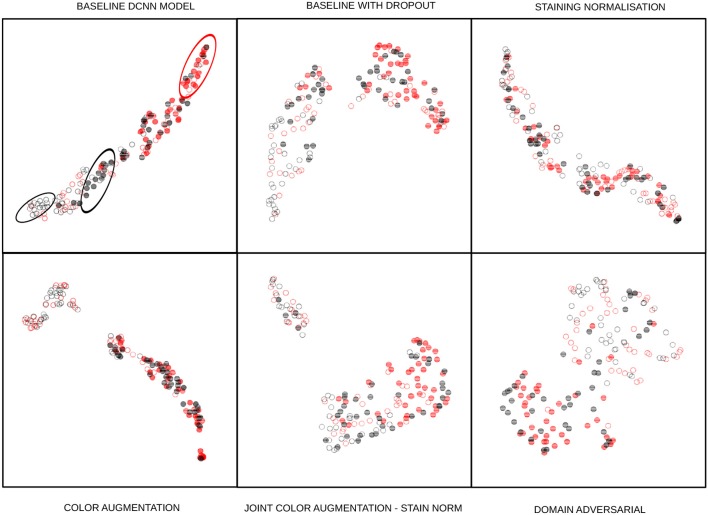
UMAP embedding of the 128-dimensional first fully connected layer features of the task branch. The points are 80 randomly sampled patches of the external test set using the baseline model with dropout: Full disks correspond to mitotic embeddings, empty circles correspond to non-mitotic ones. Red elements are from a different center than the black ones. The baseline DCNN model of the first cell shows how same-center features are clustered (ellipses), next cell shows how the baseline model with dropout drastically changes this by having a better intra-class variability than the baseline feature embeddings, presumably linked to the regularization effect induced by dropout. Staining normalization alone shows an inter-class mixed embedding, which depicts the possible overfitting in the training sources. Color augmentation also shows an excellent intra-class mixing while at the same time nicely separating mitosis from non-mitosis samples. There are local clusters in the non-mitotic samples that are visible. The joint color augmentation and staining normalization model display a similar behavior to color augmentation but with fewer separated inter-class embeddings. Finally, DANN embeddings show how the intra-class embeddings are mixed while retaining the inter-class separability, showing that it is feasible to learn the desired property of staining-invariant features.

The classification results are shown in Tables 3, 4. Each of the cells shows the average performance of the four DCNN initializations and standard deviation in parenthesis. Each column represents a combination of the strategies, and the first column is the performance of the base DCNN model without any staining normalization or augmentation technique. We trained the DCNN models without dropout but observed overfitting and degraded performance on the test sets. Therefore, all of our baseline DCNN include dropout with a probability of 0.25 in the layers described in section 3. The F1 score was selected as one of the performance measures to account for the class imbalance of our datasets. The other measure selected was the area under the receiver operating characteristics curve (AUC) since the binary decision threshold is not always close to 0.5 for all the models. Qualitative UMAP visualizations of feature embeddings for the external TUPAC dataset are displayed in Figure 8.

**Table 3:** Results on the TUPAC dataset.

**CNN model combinations**	**Baseline DCNN**						
Color augmentation		✓			✓	✓	✓
Staining normalization			✓		✓		✓
Domain adversarial				✓		✓	✓
Internal test set (F1-score)	0.8088 (±0.02)	**0.8117 (±0.001)**	0.7630 (±0.04)	0.6950 (±0.379)	0.7787 (±0.03)	0.6985 (±0.01)	0.6945 (±0.02)
External test set (F1-score)	0.71173 (±0.02)	0.7306 (±0.07)	0.5424 (±0.01)	**0.8236 (±0.071)**	0.5963 (±0.1)	0.6740 (±0.01)	0.5742 (±0.009)
Internal test set (AUC)	0.9596 (±0.006)	**0.9631 (±0.005)**	0.9351 (±0.001)	0.8972 (±0.011)	0.9503 (±0.01)	0.9030 (±0.002)	0.8871 (±0.02)
External test set (AUC)	0.8014 (±0.01)	0.8270 (±0.06)	0.848 (±0.075)	**0.9146 (±0.003)**	0.7925 (±0.06)	0.8446 (±0.004)	0.8255 (±0.06)

*Performance measures for the possible combinations of color augmentation, staining normalization, and DANN. The first column corresponds to the baseline DCNN without any staining normalization nor color augmentation. Numbers in bold indicate the best result for that row (performance measure)*.

**Table 4:** Results for the TCGA-TMAZ dataset.

**CNN model combinations**	**Baseline DCNN**						
Color augmentation		✓			✓	✓	✓
Staining normalization			✓		✓		✓
Domain adversarial				✓		✓	✓
Internal test set (F1-score)	0.5614 (±0.01)	0.5386 (±0.03)	0.5928 (±0.05)	0.6232 (±0.03)	**0.6761 (±0.01)**	0.6493 (±0.01)	0.6317 (±0.01)
External test set (F1-score)	0.4837 (±0.02)	0.5732 (±0.04)	0.5863 (±0.04)	0.5908 (±0.03)	**0.6222 (±0.05)**	0.5821 (±0.01)	0.5625 (±0.06)
Internal test set (AUC)	0.8155 (±0.01)	0.7429 (±0.01)	0.7391 (±0.01)	**0.8409** (±0.05)	0.7544 (±0.01)	0.7755 (±0.03)	0.7049 (±0.02)
External test set (AUC)	0.6368 (±0.01)	0.6735 (±0.06)	0.6633 (±0.01)	0.6838 (±0.01)	0.6798 (±0.02)	**0.6913 (±0.01)**	0.6712 (±0.01)

*F1-scores for the possible combinations of color augmentation, staining normalization and domain adversarial. The first column corresponds to the baseline model. Numbers in bold indicate the best result for that row (performance measure)*.

### 4.1. TUPAC

In the TUPAC dataset experiments, color augmentation shows a good performance on the internal test set, while the performance on the external test set is slightly above the baseline (Table 3). This result might be due to variance in the external test set staining that can not be captured by color augmentation alone. When both color augmentation and DANN are combined, the performance is not outstanding neither for internal nor external datasets. However, the results represent a good tradeoff, close to the best results for the external test dataset. The best results for the external dataset are obtained with the DANN approach. An interesting result, yet not fully understood, is that DANN show an essential decrease in performance on the internal test set. Staining normalization decreased the performance in both the internal and external test set images, likely due to overfitting in the training domains.

### 4.2. TCGA-TMAZ

For this dataset the best results involve color augmentation and DANN. DANN alone obtained the best AUC performance on the internal test set and DANN combined with color augmentation obtained the best AUC performance for the external test. Staining normalization and color augmentation together obtained the best F1 scores, both for the internal and external datasets.

### 4.3. Statistical Significance of the Results

Comparing the decisions of the best vs. second-best performing methods using the Wilcoxon signed-rank test yield the following results: For the TUPAC dataset, the difference between the color augmentation and DANN, were compared only for the external dataset, since the number of patches in the internal test set always led to a *p*-value of 0. In the external test set, the difference was significant (*p* < 0.05) in two out of four runs: *p* < 1.7232 * 10^−7^, 0.0723, 0.1808, and 1.8721 * 10^−9^, respectively.

For the TCGA-TMA dataset, the difference between the combination of color augmentation and staining normalization vs. the combination of DANN and color augmentation was significant (*p* < 0.05) in three out of four runs for the internal test dataset (*p* < 1.0922 * 10^−5^, 0.1919, 0.0008, 1.5824 * 10^−6^, respectively), and significant in the four runs for the external test dataset (*p* < 1.9029 * 10^−11^, 6.0386 * 10^−20^, 1.0652 * 10^−32^, 3.1229 * 10^−23^).

## 5. Discussion and Conclusions

The results show the importance of having separate evaluation datasets, as the results in only one can be misleading. Statistical significance of the results suggests that DANN and color augmentation (also in combination with staining normalization) can deal with the problem of having a limited set of centers for training deep learning models.

In the TCGA-TMAZ dataset, the performance gain of DANN+CA with respect to the baseline is considerable and shows how naively training DCNN models without any strategy to overcome staining variety is suboptimal.

The combination of color augmentation, staining normalization, and DANN training of DCNN models did not improve the results and, in some cases, obtained results below the baseline. Such behavior might be due to overfitting to the train centers because color augmentation over the normalized images accounts only for a limited range of variation (those in the training centers) and leading to domain adversarial training to not learn enough staining invariances. Similar behavior occurs when training DANN with stain-normalized images, for which the results are not reported here in the table, but were close and in some cases worse than the baseline.

To conclude, the experimental results show that staining normalization, color augmentation, and DANN methods improve DCNN generalization for classification tasks using digital pathology images. Results did not show a clear *winner* or combination strategy. Statistical significance tests of the results suggest that the use of color augmentation can alleviate color heterogeneity problems up to some extent and that DANN training of DCNN models alone or in combination with color augmentation can lead to even better results.

Designing deep learning experiments for computational pathology with images from different centers can provide meaningful insights about the performance of the classification algorithms in realistic scenarios (for instance by predicting class labels for data with the same pathology but scanned under different staining conditions). In future work, we devise a training of DANN with staining augmentation done in an end-to-end architecture, also evaluating DANN performance thoroughly, including the external set images in the fine-tunning of the model.

## Data Availability

The datasets analyzed for this study can be found in the TCGA-PRAD repository (https://portal.gdc.cancer.gov/projects/TCGA-PRAD) and the Replication Data for Automated Gleason grading of prostate cancer tissue microarrays via deep learning (doi: 10.7910/DVN/OCYCMP).

## Author Contributions

SO, MA, VA, AK, and HM conceived the study and wrote the manuscript. SO carried out the experiments.

### Conflict of Interest Statement

The authors declare that the research was conducted in the absence of any commercial or financial relationships that could be construed as a potential conflict of interest.

## Footnotes

^2^https://docs.gdc.cancer.gov/Encyclopedia/pages/TCGA_Barcode/

^3^https://github.com/Peter554/StainTools

^4^https://github.com/sebastianffx/stain_adversarial_learning
